# TLR2 in Pleural Fluid Is Modulated by Talc Particles during Pleurodesis

**DOI:** 10.1155/2012/158287

**Published:** 2012-12-10

**Authors:** Karolina Jankovicova, Katerina Kondelkova, Petr Habal, Ctirad Andrys, Jan Krejsek, Jiri Mandak

**Affiliations:** ^1^Department of Clinical Immunology and Allergy, Faculty of Medicine and University Hospital in Hradec Kralove, Charles University in Prague, Sokolska Street 581, 500 05 Hradec Kralove, Czech Republic; ^2^Department of Cardiac Surgery, Faculty of Medicine and University Hospital in Hradec Kralove, Charles University in Prague, Sokolska Street 581, 500 05 Hradec Kralove, Czech Republic

## Abstract

The aim of this study was to examine the role of TLR2 molecule in pleural space during thoracoscopic talc pleurodesis period in patients with malignant pleural effusion. We analyzed TLR2 molecule in soluble form as well as on membrane of granulocytes in pleural fluid. Pleural fluid examination was done at three intervals during pleurodesis procedure: 1st—before the thoracoscopic procedure, 2nd—2 hours after the terminating thoracoscopic procedure with talc insufflation, 3rd—24 hours after the thoracoscopic procedure. We reported significant increase of soluble TLR2 molecule in pleural fluid effusion during talc pleurodesis from preoperative value. This increase was approximately 8-fold in the interval of 24 hours. The changes on granulocyte population were quite different. The mean fluorescent intensity of membrane TLR2 molecule examined by flow cytometry on granulocyte population significantly decreased after talc exposure with comparison to prethoracoscopic density. To estimate the prognostic value of TLR2 expression in pleural fluid patients were retrospectively classified into either prognostically favourable or unfavourable groups. Our results proved that patients with favourable prognosis had more than 3-fold higher soluble TLR2 level in pleural fluid early, 2 hours after talc pleurodesis intervention.

## 1. Introduction

Pleural effusion is a frequent complication in many types of tumors [1]. Although there are more than 50 recognized causes of pleural effusion formation, malignancy, infection, heart failure, and pulmonary embolism are the most common reasons for pleural effusion development [2, 3].

Normally, the fluid volume is small in pleural space. It contains approximately 1 mL of fluid with the absence of inflammatory cells [2, 4]. Inflammatory changes can be initiated by penetration of foreign cells (tumor cells or microbes), proteins, or air as well as mechanical invasion [4]. Pleural effusions are either transudates or exudates reflecting the way of origin, detectable by biochemical analysis mostly by pleural fluid protein concentration and lactate dehydrogenase (LDH) level [3, 5]. The malignant pleural effusions are mainly of exudative character with hallmarks of an inflammatory process but exact mechanism of pleural fluid accumulation is not fully understood [4, 6, 7]. 

The treatment of malignant pleural effusion is always local and palliative [1]. This therapy is based on application of sclerosing agent into the pleural cavity to achieve a symphysis between the visceral and parietal pleura [8]. This approach is called chemical pleurodesis and to this day many substances have been tested for this aim (e.g., talc, silver nitrate, iodopovidone, doxycycline, and bleomycin) with different outcome [7, 8]. 

Talc seems to be the most effective agent, as well as both inexpensive and widely available [7–10]. Talc is hydrated magnesium silicate (chemically 3MgO, 4SiO_2_, and H_2_O) first used for pleurodesis already in 1935 [9]. The particles have usually diverse size but there is agreement that the largest talc particles (mean size 24.4 microns) are safer and do not provoke acute respiratory distress syndrome (ARDS), the principal complication of talc pleurodesis [7, 9]. 

The proper mechanism of talc pleurodesis at molecular level remains unclear despite of long-term widespread use. It has been demonstrated that successful talc pleurodesis is related to various cytokines and chemokines level variation in pleural fluid. Abundant proof of IL-8 (interleukin 8), TGF*β* (transforming growth factor *β*), and bFGF (basic fibroblast growth factor) increase has been documented by many research teams [3, 10–17]. The pleural mesothelial cells seem to be an essential homeostatic pivot in talc pleurodesis; however other cell types fluctuate in their number and status during this process [13, 14]. The rapid polymorphonuclear neutrophil influx follows the talc instillation reflecting the IL-8 rise simultaneously with procoagulant processes activation in pleural space [11, 14]. 

TLR2 (Toll-like receptor 2) belongs to TLR family, large receptor family consisting of at least 11 members with substantial role in innate immunity. Individual TLRs participate in recognizing specific microbial components. Amongst them TLR2 recognizes microbial PAMPs (pathogen-associated molecular patterns) such as cell wall peptidoglycan and lipoteichoic acid of Gram-positive bacteria or lipoarabinomannan of mycobacterial origin [18]. Further studies revealed more universal role of TLR2 in discrimination between either exogenous or endogenous danger signals, recognizing not only microbial PAMPs but also endogenous DAMPs (damage-associated molecular patterns) [19, 20]. 

Our study focuses on the examination of malignant pleural effusion fluid before and after talc insufflation with aim to reveal the putative role of TLR2 in inflammatory mechanisms responsible for symphysis induction in pleural space. 

## 2. Methods

The study was running during the period of two years at the Department of Cardiac Surgery and The Department of Clinical Immunology and Allergy of the University Hospital and Medical Faculty in Hradec Kralove, Charles University in Prague, Czech Republic. The study protocol was approved by the Ethics Committee of the University Hospital in Hradec Kralove and informed consent was obtained from all participants. 

### 2.1. Patients

47 patients (age 65.1 ± 9.2; M 29, F 18) with diagnosis of cancer of distinct origin with pleural effusion production were enrolled. The frequency of distinct cancer source was as follows: Lung—17 patients, small intestine—9 patients, breast—6 patients, malignant mesothelioma—5 patients, ovary—2 patients, thyroid—2 patients, mediastinum—1 patient, kidney—1 patient, lymph node—1 patient, neurosarcoma—1 patient, rectum—1 patient, and neuroendocrine tumor—1 patient. The admission criteria for enrollment into the study were as follows: the cytological evidence of malignant pleural effusion, the absence of infection in pleural space, at least the third thoracic puncture or drainage of given patient, and the shortening interval between thoracic punctures. Patients with high CRP plasma level, concomitant chemotherapy, corticotherapy, and NSAID administration as well as patients with anticoagulant heparin treatment were ruled out. 

### 2.2. Prognosis Evaluation

Patients were retrospectively classified into groups A (40) and B (7) according to the prognosis. The main prognostic criterion was early pleural effusion relapse or significant pleural effusion amount one month and later after talc pleurodesis proved by ultrasonographic analysis. Subsequent pleural effusion evaluation points were 3, 6, and 9 months after pleurodesis, respectively. Patients with poor effusion amount decrease and patients with rapid recurrence of effusion were grouped into prognostically unfavourable group B. This prognostic sorting was accomplished by thoracic surgeon. There was no difference in age, gender, cancer origin, and comorbidities between groups A and B by statistical analysis, though there was difference in other clinical data. Patients in unfavourable group B had lower body mass index, higher thoracoscore (The Thoracic Surgery Scoring System), higher number of punctures, and large pleural fluid volume before treatment (data not shown). 

### 2.3. Pleurodesis

The standard anesthesiological management according to the current protocol of Department of Cardiac Surgery, University Hospital in Hradec Kralove, using administration of sufentanil and propofol before the surgery, was exploited through the study. Muscular relaxation was achieved by cisatracurium. The perioperative antibiotic prophylaxis was maintained by one dose of ampicillin/sulbactam or cefuroxime administered intravenously in the case of allergy to beta-lactam antibiotics. Asbestos-free sterile talc was purchased from Fagron a.s. (Olomouc, Czech Republic). Talc particles (Mg_3_Si_4_O_10_(OH)_2,_ molecular weight 379.27) were introduced into the pleural cavity in the form of powder at the recommended dosage of 5 g. The diameter of talc particles was larger than 5 *μ*m^3^. Thoracoscopic procedure with pleural effusion drainage was performed immediately before the talc insufflation. After talc introduction the pleural fluid drainage continued until drained volume per 24 hours dropped under 150 mL. The average drainage time was 4 ± 1 days. 

### 2.4. Pleural Effusion Sampling and Immunostaining

Pleural effusion in amount of 5 mL was withdrawn into BD Vacutainer tubes (Beckton Dickinson and Company, USA) treated with lithium heparin. Three time points for sample collection were assigned: 1st—before the thoracoscopic procedure, 2nd—2 hours after terminating thoracoscopic procedure with talc insufflation, and 3rd—the day after the thoracoscopic procedure at 10 a.m. The interval between 1st and 2nd sampling points was 2.5 ± 0.25 hours, the interval between 2nd and 3rd sampling points was 23 ± 1 hours. After washing and filtration of pleural fluid by PBS, the standard immunofluorescence staining method for cell analysis using flow cytometry with monoclonal antibodies against CD45-APC/CD14-PerCP/CD282-PE was performed. Flow cytometry analysis on FACSCalibur (Becton Dickinson) was carried out. Flow cytometry data were acquired by CellQuest software (BD Bioscience, San Jose, USA). Anti-CD45 mouse monoclonal antibody (clone MEM-28) was purchased from Exbio, Prague, Czech Republic, anti-CD14 mouse monoclonal antibody (clone MØP9) was purchased from BD Bioscience, and anti-CD282 mouse monoclonal antibody (clone TLR2.3) was purchased from AbD Serotec, Kidlington, UK. Isotypic controls IgG1-APC (Exbio), IgG2b-PerCP (BD Bioscience), and IgG2a-PE (Beckman Coulter, Brea, California, USA) were used to identify nonspecific staining. The combination of light scatter characteristics (side scatter and forward scatter) and CD45 expression density enables accurate distinguishing of lymphocytes and granulocytes. The population of lymphocytes expresses CD45 molecule in high density and reveals low side scatter characteristics reflecting the granularity of cells. The population of granulocytes expresses CD45 molecule with low density and displays high side scatter characteristics. The CD14 marker is specific for the population of monocytes which are gated in combination with side scatter characteristics. The expression of CD282 was expressed as MFI instead of the percentage of positive cells. The expression of this surface molecule is continuous. The changes of MFI value are used in the cases of gradually expressed markers instead of % positive cells. 

Pleural fluid aliquots obtained by centrifugation at 300 g/10 min were frozen at −30°C for subsequent immunoassay. The enzyme linked immunosorbent assay (ELISA) for soluble TLR2 receptor (Uscn. Life Science Inc.) was carried out after thawing the samples according the manufacturer's recommendation. 

### 2.5. Statistical Analysis

Flow cytometry data were analyzed by FlowJo software (Tree Star, USA). The expression of CD282 was analysed separately in the population of monocytes and granulocytes. The expression was characterized by median fluorescence intensity (MFI) for each population. The expression of CD282 was expressed as changes in the MFI value in the postthoracoscopic period. The preoperative MFI value was considered as a baseline level. Clinical data, flow cytometry results, and ELISA results were statistically analyzed by MedCalc statistical software. The comparison between pleural effusion collection in three given intervals was assessed by paired *t*-test or by Wilcoxon test according to the normality of data. Difference between group A and group B was tested by *t*-test or Mann-Whithey test according to the normality of data. Distribution of categorical data into group A and group B was tested by Fisher exact test. Probability value of statistical significance testing was calculated on 0.05 level.

## 3. Results

The examination of pleural effusions in three distinct time points revealed marked changes in leukocytes subpopulation count. The relative number of lymphocytes significantly decreased from preoperative value 72.41% (IQR 26.97–85.57%) to postoperative value 11.54% (IQR 4.25–44.9%) specified by medians. The subsequent decrease in lymphocytes relative number continued up to the 3rd sampling point at 24 hours after talc pleurodesis. The median of relative lymphocyte number 24 hours after talc insufflation was only 0.83% (IQR 0.35–3.24%). The percentage of granulocytes followed the inverse course. The relative number of granulocytes increased from 33.07% (IQR 13.07–71.16%) preoperatively to 90.21% (IQR 60.12–95.13%) postoperatively specified by medians. The subsequent increase in granulocytes relative number continued up to the 3rd sampling point at 24 hours after pleurodesis when median of relative granulocyte number reached 97.81% (IQR 92.16–99.25%). Monocytes were detected only in a very small amount in all three sampling points. They represented 1.35% (IQR 0.4–2.55%) in preoperative sampling point, decreased to nearly 0% (IQR 0–0.78%) 2 hours postoperatively, and represented 0.63% (IQR 0.17–2.47%) 24 hours postoperatively specified by medians. There was no difference in percentage of leukocyte subpopulations between prognostically favourable group A and prognostically unfavourable group B (data not shown). 

**Figure 1:**
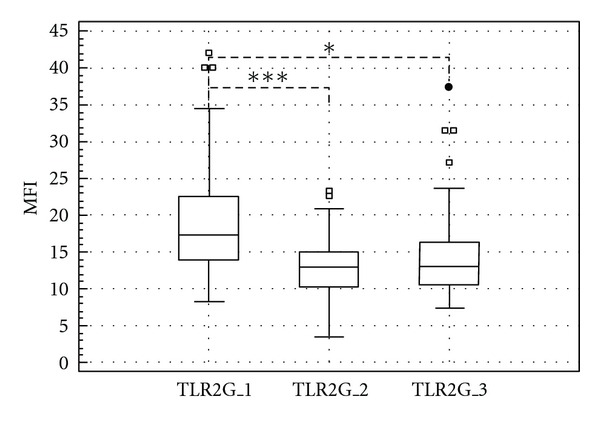
The mean fluorescence intensity (MFI) of TLR2 on membrane of granulocytes in pleural effusion fluid at three sampling points: 1—before thoracoscopic procedure, 2—2 hours after terminating talc insufflation procedure, 3—24 hours after talc insufflation procedure. (Line: median; boxes: quartiles; whiskers: nonoutliers range.) Significance: *P = 0,05–0,01; **P = 0,01–0,001; ***P < 0,001.

The expression of TLR2 was monitored on membrane of granulocytes as well as in pleural fluid in its soluble form. The membrane changes in granulocyte population were statistically significant in two intervals between pleural samples collections. Both postthoracoscopic sampling of pleural fluid showed decreased density of membrane TLR2 with comparison to preoperative density on granulocytes. The MFI of membrane TLR2 expression on granulocytes dropped from 17.34 (IQR 13.73–22.58) preoperatively to 12.88 (IQR 10.07–14.92) 2 hours after talc pleurodesis procedure, specified by medians (*P* < 0.001). There was also difference between preoperative MFI of membrane TLR2 expression on granulocytes and its MFI 24 hours after talc pleurodesis procedure that represented by median 13.08 (IQR 10.42–16.27) (*P* < 0.05) (Figure 1). No difference in granulocyte TLR2 expression between two prognostic groups of patients was found. 

**Figure 2:**
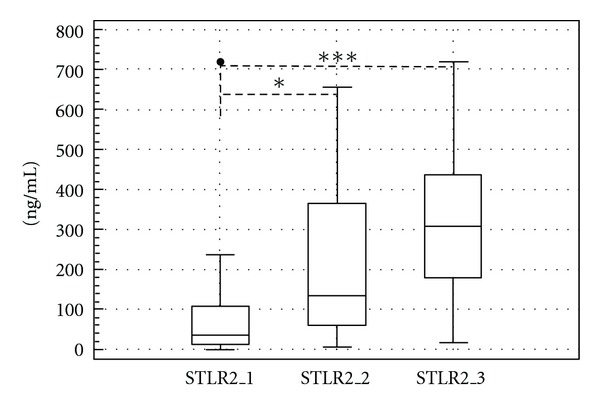
The concentration of soluble TLR2 (ng/mL) in pleural effusion fluid at three sampling points: 1—before thoracoscopic procedure, 2—2 hours after terminating talc insufflation procedure, 3—24 hours after talc insufflation procedure. (Line: median; boxes: quartiles; whiskers: nonoutliers range.) Significance: *P = 0,05–0,01; **P = 0,01–0,001; ***P < 0,001.

**Figure 3:**
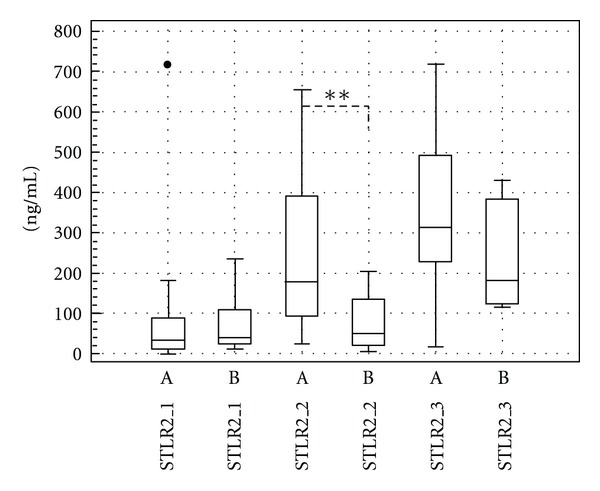
The concentration of soluble TLR2 (ng/mL) in pleural effusion fluid at three sampling points in prognostic favourable group of patients (A) and prognostic unfavourable group of patients (B): 1—before thoracoscopic procedure, 2—2 hours after terminating talc insufflation procedure, 3—24 hours after talc insufflation procedure. (Line: median; boxes: quartiles; whiskers: nonoutliers range.) Significance: *P = 0,05–0,01; **P = 0,01–0,001; ***P < 0,001.

The changes in soluble form of TLR2 were more pronounced than changes in membrane TLR2 density on granulocyte population. The multiple increase in soluble TLR2 concentration in pleural fluid after talc insufflation has been revealed. The production of soluble TLR2 rose from 37 ng/mL (IQR 11.75–108.25 ng/mL) preoperatively to 132 ng/mL (IQR 54.75–367.25 ng/mL) 2 hours after talc pleurodesis procedure (*P* < 0.05) and to 309 ng/mL (IQR 162–436 ng/mL) 24 hours after talc pleurodesis procedure (*P* < 0.001), specified by medians (Figure 2). These changes were found significantly different between both prognostic groups of patients 2 hours after talc pleurodesis procedure. The patients with favourable course had substantially higher pleural concentration of soluble TLR2 (median 180 ng/mL, IQR 92–400.5 ng/mL) than patients with unfavourable prognosis (median 50 ng/mL, IQR 19–138 ng/mL) (*P* < 0.01). This difference seems to continued to the 3rd sampling time point at 24 hours after talc pleurodesis procedure but without statistical significance (Figure 3). In patients with detectable monocyte population TLR2 expression on monocytes was approximately 10-fold higher than in granulocyte population (data not shown). 

## 4. Discussion

The pleural fluid is physiologically produced in a very small amount with the constitution biochemically similar to plasma filtrate [21]. Despite of minor content of pleural fluid normally formed there is an active circulation between interpleural space and surrounding space through pleural blood and lymphatic capillary system and mesothelial cells permeability ensuring approximately 250 mL fluid recovery per 24 hours [22]. This natural fluid permutation can be affected by local inflammation or hydrostatic disbalance during cancer-related inflammatory response [3, 23]. The pleural mesothelial cells are the key homeostatic regulators in pleural space. They express multiple pattern recognition receptors and trigger acute inflammatory reaction often by danger signal recognition. In physiologic conditions there is only negligible contribution of other inflammatory cells but after any inflammatory stimulus immunocompetent cells can rapidly move into the pleural space [4, 16]. 

To date chemical pleurodesis by talc is the optimal treatment option for malignant pleural effusion [24]. It has been demonstrated that talc particles directly induce mesothelial cells to produce inflammatory cytokines, mainly IL-8, VEGF (vascular endothelial growth factor), and MCP-1 (monocyte chemotactic protein-1) [13, 14, 16]. These proinflammatory changes are followed by rapid polymorphonuclear neutrophils influx into the pleural space [11]. We observed this trend in our study as the relative number of polymorphonuclear neutrophils increased from 33.07% preoperatively to 90.21% after talkage. There is also evidence that talc enhances intercellular adhesion molecule-1 (ICAM-1) expression on pleural mesothelial cells [13]. Pleural mesothelial cells treated by talc secrete bFGF (basic fibroblast growth factor) and TGF*β* (transforming growth factor *β*) responsible for fibroblast activation, and enhanced fibrogenesis including collagen production ultimating in effective pleural fibrosis [10, 15].

Regardless of central initiating role of pleural mesothelial cells in the process of talc pleurodesis, relatively a little is known about the role and pathophysiological contribution of other cell types present in malignant pleural effusion. It is known that neutrophil-rich fluid is a hallmark of an acute inflammatory process, whereas a lymphocyte-predominant fluid profile suggests a chronic process induced by, for example, cancer [3]. Our study proved this fact, as we found 72.41% of lymphocytes in malignant pleural effusion fluid before talc treatment. There is no published study yet aiming the TLR2 expression in pleural fluid in relation to inflammatory changes provoked by talc particles. Most studies investigating pleural fluid analyzed expression of TLR2 in the course of microbial infections. TLR2 has the capability to recognize the broadest range of PAMPs among all TLR receptors [25]. This receptor is predominantly expressed on cells involved in the first-line host defense, including monocytes, macrophages, dendritic cells, and neutrophils. Lower expression is observed on both endothelial and epithelial cells. Fan showed that TLR2 upregulation in alveolar macrophages resulted in enhanced cytokine expression and neutrophils migration [26]. Chen et al. demonstrated that TLR2 molecule is expressed on CD4+ T cell in pleural fluid in patients with tuberculous pleurisy and it probably mediate direct interaction between M. *tuberculosis* and CD4+ T cell as a costimulatory receptor [27]. The mesothelial cells can be potential source of TLR2 as well. Hussain et al. showed TLR2 receptor expression and its upregulation after exposure to staphylococcal peptidoglycan on murine pleural mesothelial cells [28]. The differential expression of some TLRs in pleural fluid comparing transudative, infectious, and malignant pleural effusions on both mRNA and protein level has been outlined. These differences were not established for TLR2 molecule [29, 30]. Fan et al. concluded that TLR2 is positioned at the interface of microbial and sterile inflammation by selectively responding to both bacterial products and endogenous danger ligands (DAMPs) including hyaluronic acid, heparan sulfate, fibrinogen, heat shock proteins, and high-mobility group box 1 protein (HMGB1) [31]. The endogenous formation of crystals such as urates or pyrophosphates is followed by an intensive inflammatory response as these crystals serve as DAMPs. It has recently been revealed that inorganic aluminium hydroxide used as an adjuvant for decades is also serving as a danger signal for innate immunity [32]. The TLR2 signaling on mesothelial cells may be the important inducible mechanism activating the innate immune system after tissue and cell damage. In our study we demonstrated rapid increase of TLR2 concentration in pleural fluid after talc insufflation. The increase was approximately 8-fold in the interval of 24 hours. Prognostically interesting information is that patients with subsequent beneficial history had more than 3-fold higher soluble TLR2 concentration already 2 hours after talc pleurodesis intervention in comparison with patients with poor prognosis. Although the membrane origin of this soluble TLR2 molecule is not clear, pleural mesothelial cells seem to be putative candidate for TLR2 production. It is known that multipotent mesothelial cells ensure primary response to noxious agents such as talc. Talc insufflation has been noted to stimulate pleural mesothelial cells to release endostatin, an inhibitor of angiogenesis. Talc appears to alter among others angiogenic balance in the pleural space from proangiogenic environment induced by malignant cells proliferation to more angiostatic one [4]. It has been published that another sclerosing agent was used to induce pleural symphysis, bleomycin, interact with TLR2 receptor on human monocytic cell line THP1, and stimulate cells to produce proinflammatory cytokines [33]. It is known from several studies that talc particles induce granulomas *in vivo *like other particles that are poorly degraded by phagocytic cells [34]. *In vitro*, talc particles inhibit lymphocyte proliferation and antibody production and display cytotoxic effect on macrophages [35, 36]. In our study only minor contribution of monocyte/macrophage cell population in pleural fluid was detected. Macrophage population is probably adhered to pleural layer and is not released to pleural fluid effusion in our interpretation. 

The expression of TLR2 molecule on polymorphonuclear granulocytes was studied with respect to infection, sepsis, surgical stress, or other medical intervention. Granulocytes downregulate TLR2 expression in response to LPS, GM-CSF, and TNF cytokines *in vitro*. This early TLR2 downregulation may be the consequence of the externalization of entire molecule rather than transcriptional regulation of TLR2 biosynthesis [37, 38]. TLR2 molecule is downmodulated after interaction with cellulose acetate beads, a medical apheretic device used for treatment of inflammatory diseases [39]. Intensity of TLR2 expression on granulocytes is also significantly reduced during cardiopulmonary bypass surgery [40]. Our study proved downregulation of TLR2 expression on granulocytes during talc pleurodesis. However, we can only speculate about the direct effect of talc particles on granulocytes, cytokine influence, or combination of both variables. 

## 5. Conclusions

In conclusion, we report the significant changes of TLR2 molecule in pleural fluid effusion during talc pleurodesis as in its soluble form as on membrane of granulocytes. Relatively early after talc pleurodesis intervention prognostically favourable group of patients displays more than 3-fold higher soluble TLR2 level in pleural fluid compared to unfavourable group. It remains to be determined by further studies what is the cell origin of TLR2 to better understand the complex mechanism of talc induced inflammatory changes in the pleural space. 

## References

[1] Lombardi G, Zustovich F, Nicoletto MO, Donach M, Artioli G, Pastorelli D (2010). Diagnosis and treatment of malignant pleural effusion: a systematic literature review and new approaches. *American Journal of Clinical Oncology*.

[2] Idris L, Ranaweera N, Laws D (2011). Investigation of pleural effusions. *Acute Medicine*.

[3] Porcel JM, Light RW (2006). Diagnostic approach to pleural effusion in adults. *American Family Physician*.

[4] Jantz MA, Antony VB (2008). Pathophysiology of the pleura. *Respiration*.

[5] Huggins JT, Doelken P, Sahn SA (2010). The unexpandable lung. *F1000 Medicine Reports*.

[6] Porcel JM (2011). Pearls and myths in pleural fluid analysis. *Respirology*.

[7] Uzbeck MH, Almeida FA, Sarkiss MG (2010). Management of malignant pleural effusions. *Advances in Therapy*.

[8] Rodriguez-Panadero F, Montes-Worboys A (2012). Mechanisms of pleurodesis. *Respiration*.

[9] Janssen JP, Collier G, Astoul P (2007). Safety of pleurodesis with talc poudrage in malignant pleural effusion: a prospective cohort study. *The Lancet*.

[10] Antony VB, Nasreen N, Mohammed KA (2004). Talc pleurodesis: basic fibroblast growth factor mediates pleural fibrosis. *Chest*.

[11] van den Heuvel MM, Smit HJM, Barbierato SB, Havenith CEG, Beelen RHJ, Postmus PE (1998). Talc-induced inflammation in the pleural cavity. *European Respiratory Journal*.

[12] Gary Lee YC, Melkerneker D, Thompson PJ, Light RW, Lane KB (2002). Transforming growth factor *β* induces vascular endothelial growth factor elaboration from pleural mesothelial cells in vivo and in vitro. *American Journal of Respiratory and Critical Care Medicine*.

[13] Nasreen N, Hartman DL, Mohammed KA, Antony VB (1998). Talc-induced expression of C-C and C-X-C chemokines and intercellular adhesion molecule-1 in mesothelial cells. *American Journal of Respiratory and Critical Care Medicine*.

[14] Montes-Worboys A, Rodriguez-Portal JA, Arellano-Orden E, Digón-Pereiras J, Rodriguez-Panadero F (2010). Interleukin-8 activates coagulation and correlates with survival after talc pleurodesis. *European Respiratory Journal*.

[15] Marchi E, Vargas FS, Acencio MM, Antonangelo L, Genofre EH, Teixeira LR (2006). Evidence that mesothelial cells regulate the acute inflammatory response in talc pleurodesis. *European Respiratory Journal*.

[16] Acencio MMP, Vargas FS, Marchi E (2007). Pleural mesothelial cells mediate inflammatory and profibrotic responses in talc-induced pleurodesis. *Lung*.

[17] Idell S (2008). The pathogenesis of pleural space loculation and fibrosis. *Current Opinion in Pulmonary Medicine*.

[18] Takeda K, Akira S (2005). Toll-like receptors in innate immunity. *International Immunology*.

[19] Erridge C (2010). Endogenous ligands of TLR2 and TLR4: agonists or assistants?. *Journal of Leukocyte Biology*.

[20] Piccinini AM, Midwood KS (2010). DAMPening inflammation by modulating TLR signalling. *Mediators of Inflammation*.

[21] Tyan YC, Wu HY, Su WC, Chen PW, Liao PC (2005). Proteomic analysis of human pleural effusion. *Proteomics*.

[22] Mayer KK (1958). Direct lymphatic connections from the lower lobes of the lung to the abdomen. *The Journal of Thoracic Surgery*.

[23] Kaczmarek M, Nowicka A, Kozłowska M, Zurawski J, Batura-Gabryel H, Sikora J (2011). Evaluation of the phenotype pattern of macrophages isolated from malignant and non-malignant pleural effusions. *Tumor Biology*.

[24] Zahid I, Routledge T, Bille A, Scarci M (2011). Best evidence topic-thoracic oncologic: what is the best treatment for malignant pleural effusions?. *Interactive Cardiovascular and Thoracic Surgery*.

[25] Borrello S, Nicolò C, Delogu G, Pandolfi F, Ria F (2011). TLR2: a crossroads between infections and autoimmunity?. *International Journal of Immunopathology and Pharmacology*.

[26] Fan J (2010). TLR cross-talk mechanism of hemorrhagic shock-primed pulmonary neutrophil infiltration. *The Open Critical Care Medicine Journal*.

[27] Chen X, Zhang M, Zhu X (2009). Engagement of toll-like receptor 2 on CD4+ T cells facilitates local immune responses in patients with tuberculous pleurisy. *Journal of Infectious Diseases*.

[28] Hussain T, Nasreen N, Lai Y, Bellew BF, Antony VB, Mohammed KA (2008). Innate immune responses in murine pleural mesothelial cells: toll-like receptor-2 dependent induction of *β*-defensin-2 by staphylococcal peptidoglycan. *American Journal of Physiology-Lung Cellular and Molecular Physiology*.

[29] Chang LC, Hua CC, Chu CM, Chiang BY, Chen HJ, Yu CC (2009). Differential mRNA expression of Toll-like receptors and their adaptors in pleural effusions. *Respirology*.

[30] Yang HB, Xie KQ, Deng JM, Qin SM (2010). Expression of soluble toll-like receptors in pleural effusions. *Chinese Medical Journal*.

[31] Fan J, Xiang M, Fan J (2010). Association of toll-like receptor signaling and reactive oxygen species: a potential therapeutic target for posttrauma acute lung injury. *Mediators of Inflammation*.

[32] Lambrecht BN, Kool M, Willart MA, Hammad H (2009). Mechanism of action of clinically approved adjuvants. *Current Opinion in Immunology*.

[33] Razonable RR, Henault M, Paya CV (2006). Stimulation of toll-like receptor 2 with bleomycin results in cellular activation and secretion of pro-inflammatory cytokines and chemokines. *Toxicology and Applied Pharmacology*.

[34] Radic I, Vucak I, Milosevic J, Marusic A, Vukicevic S, Marusic M (1988). Immunosuppression induced by talc granulomatosis in the rat. *Clinical and Experimental Immunology*.

[35] Davies R, Skidmore JW, Griffiths DM, Moncrieff CB (1983). Cytotoxicity of talc for macrophages in vitro. *Food and Chemical Toxicology*.

[36] Hoffeld JT (1983). Inhibition of lymphocyte proliferation and antibody production in vitro by silica, talc, Bentonite or Corynebacterium parvum: involvement of peroxidative processes. *European Journal of Immunology*.

[37] Flo TH, Halaas O, Torp S (2001). Differential expression of Toll-like receptor 2 in human cells. *Journal of Leukocyte Biology*.

[38] Acorci-Valério MJ, Bordon-Graciani AP, Dias-Melicio LA, de Assis Golim M, Nakaira-Takahagi E, de Campos Soares AM (2010). Role of TLR2 and TLR4 in human neutrophil functions against paracoccidioides brasiliensis. *Scandinavian Journal of Immunology*.

[39] Hidaka M, Fukuzawa K (2011). Down-modulation of toll-like receptor 2 expression on granulocytes and suppression of interleukin-8 production due to in vitro treatment with cellulose acetate beads. *Therapeutic Apheresis and Dialysis*.

[40] Krejsek J, Kunes P, Kolackova M (2008). Expression of Toll-like receptors 2 and 4 on innate immunity cells modulated by cardiac surgical operation. *Scandinavian Journal of Clinical and Laboratory Investigation*.

